# Development and validation of a nomogram predictive model for cognitive impairment in cerebral small vessel disease: a comprehensive retrospective analysis

**DOI:** 10.3389/fneur.2024.1373306

**Published:** 2024-06-17

**Authors:** Ning Li, Yan Gao, Li-tao Li, Ya-dong Hu, Li Ling, Nan Jia, Ya-jing Chen, Ya-nan Meng, Ye Jiang

**Affiliations:** ^1^Department of Neurology, Affiliated Hospital of Hebei University, Baoding, China; ^2^Department of Neurology, Renmin Hospital of Wuhan University, Wuhan, China; ^3^Department of Neurology, Hebei General Hospital, Shijiazhuang, China

**Keywords:** cerebral small vessel disease, cognitive impairment, predictive modeling, LASSO regression, logistic regression, nomogram

## Abstract

**Background:**

Cerebral small vessel disease (CSVD) is a common neurodegenerative condition in the elderly, closely associated with cognitive impairment. Early identification of individuals with CSVD who are at a higher risk of developing cognitive impairment is crucial for timely intervention and improving patient outcomes.

**Objective:**

The aim of this study is to construct a predictive model utilizing LASSO regression and binary logistic regression, with the objective of precisely forecasting the risk of cognitive impairment in patients with CSVD.

**Methods:**

The study utilized LASSO regression for feature selection and logistic regression for model construction in a cohort of CSVD patients. The model’s validity was assessed through calibration curves and decision curve analysis (DCA).

**Results:**

A nomogram was developed to predict cognitive impairment, incorporating hypertension, CSVD burden, apolipoprotein A1 (ApoA1) levels, and age. The model exhibited high accuracy with AUC values of 0.866 and 0.852 for the training and validation sets, respectively. Calibration curves confirmed the model’s reliability, and DCA highlighted its clinical utility. The model’s sensitivity and specificity were 75.3 and 79.7% for the training set, and 76.9 and 74.0% for the validation set.

**Conclusion:**

This study successfully demonstrates the application of machine learning in developing a reliable predictive model for cognitive impairment in CSVD. The model’s high accuracy and robust predictive capability provide a crucial tool for the early detection and intervention of cognitive impairment in patients with CSVD, potentially improving outcomes for this specific condition.

## 1 Introduction

Cerebral small vessel disease (CSVD) is intricately linked to cognitive decline and represents a critical focus in the study of vascular contributions to cognitive impairment and dementia (1–3). This spectrum of pathological processes has been increasingly recognized for its substantial impact on public health, especially given the aging global population (2, 4–7). Despite its clinical importance, the detection and quantification of cognitive impairment attributable to CSVD remain fraught with complexities. Cognitive symptoms often manifest subtly and progress insidiously, making early diagnosis a formidable challenge in clinical settings (8).

The traditional approach to diagnosing CSVD-related cognitive impairment relies heavily on clinical judgment, which is subject to variability and may fail to capture the nuanced progression of the disease. Consequently, there is a pressing need for objective and reproducible diagnostic tools that can accurately predict the onset and trajectory of cognitive decline in CSVD. Addressing this need, our study introduces a predictive model that synthesizes demographic, clinical, neuroimaging, and biomarker data to objectively assess the risk of cognitive decline. The current models in literature have either not incorporated such a diverse dataset or have not been validated sufficiently for clinical application (9, 10). Our approach utilizes advanced machine learning techniques, including LASSO regression for feature selection and logistic regression for model development, filling a critical gap by providing a tool with both high sensitivity and specificity for CSVD cognitive sequelae.

This study leverages a comprehensive dataset of 377 CSVD patients, encompassing a wide array of variables including demographic details, clinical history, neuroimaging findings, and biomarkers. Through rigorous statistical methodologies, we aim to construct a predictive model that not only distinguishes between patients with and without cognitive impairment but also provides a probabilistic assessment of the risk of cognitive decline. By adopting machine learning techniques, particularly LASSO regression for feature selection followed by logistic regression for model development, we seek to create a model that is both sensitive and specific to the cognitive sequelae of CSVD.

In doing so, our objectives are twofold: firstly, to present a model that can be readily applied in clinical practice for the early identification of patients at risk of cognitive impairment due to CSVD, and secondly, to contribute to the body of knowledge that underpins the intersection of neurovascular pathology and neurodegeneration. It is our anticipation that such a model will not only facilitate early intervention strategies but also spur further research into targeted therapies for this underdiagnosed yet prevalent condition.

## 2 Materials and methods

### 2.1 Study population and design

In a cross-sectional study conducted from July 2022 to October 2023 at the Neurology Department of the Affiliated Hospital of Hebei University, we targeted a cohort of patients aged 50 and above. These participants were subjected to comprehensive cranial magnetic resonance imaging (MRI) to confirm CSVD diagnosis. The inclusion criteria mandated completion of a full cranial MRI series, provision of serum samples for ELISA testing to assess inflammatory markers, and undergoing the Montreal Cognitive Assessment (MoCA) to gauge cognitive function. The MoCA, a widely acknowledged tool for detecting mild cognitive impairments, was specifically chosen for its robust validity across diverse age groups and clinical conditions (11, 12), thereby serving as an optimal instrument for assessing CSVD-related cognitive impairments. The scoring of MoCA was conducted by trained personnel, adhering to standardized procedures to ensure consistency and reliability of cognitive function assessment. Exclusion criteria encompassed the presence of neurological conditions other than CSVD that might impair cognitive function, a history of psychiatric disorders or current use of psychoactive drugs, contraindications to MRI such as claustrophobia or presence of metal implants, inadequate quality of cranial MRI for reliable assessment, acute infections and severe systemic illnesses that could impede participation in the study. Participants’ cognitive status was categorized based on MoCA scores, with scores of 25 or below indicating cognitive impairment, and scores of 26 or above denoting normal cognitive function. Alongside MRI diagnostics, participants underwent routine lab tests including complete blood count, renal and liver function tests, and electrolyte panels. Informed consent for serum sample collection for ELISA testing was obtained in line with ethical standards. This study was conducted in strict adherence to ethical guidelines and was approved by the Ethics Committee of the Affiliated Hospital of Hebei University, with the approval number HDFYLL-KY-2023-060. This approval ensures that our study complies with the ethical principles outlined in the 1964 Helsinki Declaration and its subsequent amendments, reaffirming our commitment to the highest standards of research ethics.

### 2.2 MRI acquisition and assessment in CSVD patients

In our study, all participants were subjected to brain MRI examinations using a state-of-the-art 3.0 T GE scanner. The identification of CSVD was reliant on the detection of specific neuroimaging indicators, which included any combination of lacunes, white matter hyperintensities (WMH), enlarged perivascular spaces (EPVS), and cerebral microbleeds (CMBs). In this context, WMH were defined as regions showing elevated signal intensity on T2-weighted images, often symmetrically distributed across brain hemispheres. Lacunes were described as round or ovoid cerebrospinal fluid-signal lesions with diameters ranging between 3 and 15 mm. Additionally, CMBs manifested as small, round, hypointense areas, 2–10 mm in size, evident in susceptibility-weighted imaging sequences. In line with the comprehensive CSVD scoring methodology initially developed by Wardlaw et al. (5, 11), we evaluated the total CSVD burden employing an ordinal scale that spans from 0 to 4. This evaluation process included assigning one point for each of the following criteria: the severity of WMH, as indicated by a periventricular WMH score of 3 or a deep WMH score between 2 and 3; the presence of lacunes; the existence of CMBs; and the significant presence of basal ganglia EPVS, particularly exceeding a count of 10 (12). For an accurate and unbiased evaluation of these CSVD markers, neuroimaging specialists Yan Hou and Huan Zhou, devoid of access to the participants’ clinical information, undertook the assessment. Their analysis adhered rigorously to the Standards for Reporting Vascular Changes on Neuroimaging (STRIVE) criteria (11, 13), which provided a framework for consistent and reliable interpretation of CSVD-related neuroimaging findings.

### 2.3 Clinical blood biochemistry assessment

In this study, we performed an extensive assessment of clinical blood biochemistry parameters in patients with CSVD. Our evaluation included a broad spectrum of 44 laboratory markers, categorically divided into routine and specialized tests. Routine assessments comprised complete blood count, renal and liver function tests, electrolyte balance, lipid profiles, and coagulation parameters. In addition to these standard measures, we conducted a detailed analysis of inflammatory markers, crucial in elucidating CSVD’s pathophysiological landscape. These markers encompassed cytokines, acute phase reactants, and specific inflammatory indices, offering insights into the inflammatory status of CSVD patients. Notably, all laboratory data were transformed into binary categorical variables, based on either their median values or clinically established cutoffs. This transformation enabled a nuanced exploration of the potential correlations between these biochemical markers and cognitive impairment in CSVD, forming a critical component of our predictive model development.

### 2.4 Clinical evaluation

In this study, we conducted a thorough clinical evaluation of 377 patients diagnosed with CSVD. The participants’ demographic information, including age and sex, was meticulously recorded. Medical histories were detailed, focusing on the presence of hypertension, diabetes, and hypercholesterolemia. Hypertension was defined as having a systolic blood pressure ≥140 mmHg, diastolic blood pressure ≥90 mmHg, or being under antihypertensive medication. Diabetes was identified by fasting blood glucose levels ≥7.0 mmol/L, OGTT2h levels ≥11.1 mmol/L, or the use of hypoglycemic medications. Hypercholesterolemia was recognized by total cholesterol or LDL cholesterol levels exceeding normal range limits. Additionally, lifestyle factors such as smoking and alcohol consumption histories were gathered, alongside information on the duration of the disease.

### 2.5 Statistical methodology

In our investigation involving 377 CSVD patients, the cohort was randomly divided into a training set with 265 participants and a validation set comprising 112 participants, following a 7:3 allocation ratio. This randomization ensured a balanced and unbiased approach to model development. we opted for the stratification of continuous laboratory variables into tertiles. This approach was employed to facilitate a more meaningful interpretation of the data by categorizing it into low, medium, and high ranges. Utilizing tertiles allows for a clear delineation of patient subgroups based on their laboratory values, aligning with the clinical relevance of these categories. The selection of thresholds for continuous variables was informed by clinical expertise and mirrored established norms in contemporary research and statistical methodologies. For categorical data, we presented frequencies and percentages. To ascertain baseline similarities or disparities between the training and validation cohorts, we utilized appropriate statistical tests, opting for the *χ*^2^ test or Fisher’s exact test for categorical data.

Within the training dataset, the LASSO regression method was employed to pinpoint crucial risk factors linked to cognitive impairment in CSVD patients. LASSO regression was utilized for its efficacy in both variable selection and regularization, enabling the identification of the most predictive features for cognitive impairment in CSVD while mitigating overfitting. This technique helped in selecting variables with non-zero coefficients for further analysis. These identified variables were then incorporated into a logistic regression model to ascertain independent predictors of cognitive impairment in CSVD. We constructed a nomogram from these identified risk factors, derived from the logistic regression model. The nomogram’s predictive performance was evaluated using the receiver operating characteristic curve (AUC-ROC), and calibration curves were generated to align predicted probabilities with actual outcomes. Furthermore, the clinical utility of the model was assessed using decision curve analysis (DCA). To mitigate overfitting, we employed several strategies. First, we used LASSO regression to perform variable selection and reduce model complexity. Second, we conducted 10-fold cross-validation to determine the optimal regularization parameter (*λ*), ensuring the model’s performance on multiple data subsets. Finally, we validated the model using an independent dataset to test its generalizability. These combined strategies enhance the model’s robustness and reduce the risk of overfitting. All statistical procedures were carried out using R software (version 4.3.0),^1^ and a *p*-value of less than 0.05 (two-tailed) was considered to denote statistical significance.

## 3 Results

### 3.1 Baseline characteristics

**Figure 1:**
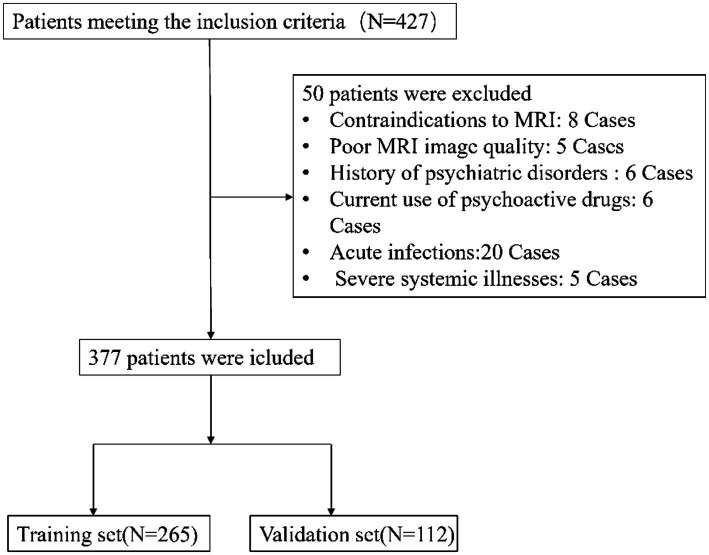
Patient selection flowchart. This diagram details the process of selecting eligible CSVD patients for the study, highlighting the inclusion and exclusion criteria. It shows the initial number of participants, the application of exclusion criteria, and the final number of patients included in the analysis.

From July 2022 to October 2023, this investigation initially recruited 427 participants who satisfied the inclusion benchmarks. Subsequent application of exclusion criteria led to the withdrawal of 50 participants, yielding a dataset of 377 individuals for subsequent analyses as depicted in Figure 1. Of these, 265 formed the training cohort, while the remaining 112 were allocated to the validation cohort. Table 1 delineates the demographic and clinical profiles at baseline for both cohorts. This study incorporated the following 52 potential indicators related to cognitive impairment in CSVD: CSVD Burden, Gender, Smoking, Drinking, Hypertension, Diabetes, Hyperlipidemia, Age, White Blood Cell Count, Platelet Count, Neutrophil Count, Lymphocyte Count, Monocyte Count, Urea, Creatinine, Uric Acid, Prothrombin Time, Activated Partial Thromboplastin Time, Thrombin Time, Fibrinogen, Hemoglobin A1c, Total Cholesterol, Triglycerides, High-Density Lipoprotein, Low-Density Lipoprotein, Very Low-Density Lipoprotein, Apolipoprotein A1 (ApoA1), Apolipoprotein B100, ApoA1 to ApoB100 Ratio, Lipoprotein(a), Total Protein, Albumin, Globulin, Albumin to Globulin Ratio, Homocysteine, Systemic Immune-Inflammatory Index, Systemic Inflammation Response Index, Neutrophil-to-Lymphocyte Ratio, Lymphocyte-to-Monocyte Ratio, Neutrophil-to-Monocyte Ratio, Neutrophil-to-HDL Ratio, Lymphocyte-to-HDL Ratio, Monocyte-to-HDL Ratio, IL-6, TNFα, VCAM-1, LP-PLA2, CD40L, E-Selectin, ADMA, vWF, and ICAM-1. The lack of significant disparities in the 52 evaluated variables between the training and validation sets underscores the homogeneity of the study population. This parity is critical as it suggests that the predictive model derived from the training set has the potential for generalizability to other patient cohorts, affirming the robustness of the model’s predictive capacity for cognitive impairment associated with CSVD (Table 1).

**Table 1:** Comparative analysis of potential predictive factors in a cerebral small vessel disease (CSVD) cognitive impairment predictive model between training and validation sets.

Variables	Total (*n* = 377)	training set (*n* = 265)	validation set (*n* = 112)	*p*-value
Cognitive impairment, *n* (%)				0.438
No	132 (35)	89 (34)	43 (38)	
Yes	245 (65)	176 (66)	69 (62)	
CSVD.Burden, *n* (%)				0.912
0–1	173 (46)	120 (45)	53 (47)	
2	89 (24)	64 (24)	25 (22)	
3–4	115 (31)	81 (31)	34 (30)	
Gender, *n* (%)				0.273
Female	183 (49)	134 (51)	49 (44)	
Male	194 (51)	131 (49)	63 (56)	
Smoking, *n* (%)				0.8
No	261 (69)	185 (70)	76 (68)	
Yes	116 (31)	80 (30)	36 (32)	
Drinking, *n* (%)				0.174
No	124 (33)	81 (31)	43 (38)	
Yes	253 (67)	184 (69)	69 (62)	
Hypertension, *n* (%)				0.3
No	103 (27)	77 (29)	26 (23)	
Yes	274 (73)	188 (71)	86 (77)	
Diabetes, *n* (%)				0.3
No	267 (71)	183 (69)	84 (75)	
Yes	110 (29)	82 (31)	28 (25)	
Hyperlipidemia, *n* (%)				0.956
No	250 (66)	175 (66)	75 (67)	
Yes	127 (34)	90 (34)	37 (33)	
Age, *n* (%)				0.9
≤69	249 (66)	174 (66)	75 (67)	
>69	128 (34)	91 (34)	37 (33)	
White blood cell count (×10^9^/L), *n* (%)				0.842
<6.04	126 (33)	91 (34)	35 (31)	
6.04–7.70	128 (34)	89 (34)	39 (35)	
>7.70	123 (33)	85 (32)	38 (34)	
Platelet count (×10^9^/L), *n* (%)				0.973
<200	126 (33)	89 (34)	37 (33)	
200–250	128 (34)	89 (34)	39 (35)	
>250	123 (33)	87 (33)	36 (32)	
Neutrophil count (×10^9^/L), *n* (%)				0.581
<3.83	127 (34)	86 (32)	41 (37)	
3.83–5.34	125 (33)	92 (35)	33 (29)	
>5.34	125 (33)	87 (33)	38 (34)	
Lymphocyte count (×10^9^/L), *n* (%)				0.533
<130	126 (33)	93 (35)	33 (29)	
1.30–1.74	126 (33)	85 (32)	41 (37)	
>1.74	125 (33)	87 (33)	38 (34)	
Monocyte count (×10^9^/L), *n* (%)				0.612
<0.40	128 (34)	87 (33)	41 (37)	
0.40–0.54	128 (34)	94 (35)	34 (30)	
>0.54	121 (32)	84 (32)	37 (33)	
Urea (mmol/L), *n* (%)				0.791
<5.00	120 (32)	87 (33)	33 (29)	
5.00–6.19	120 (32)	84 (32)	36 (32)	
>6.19	137 (36)	94 (35)	43 (38)	
Creatinine (μmol/L), *n* (%)				0.482
<57	128 (34)	88 (33)	40 (36)	
57–70	128 (34)	95 (36)	33 (29)	
>70	121 (32)	82 (31)	39 (35)	
Uric acid (μmol/L), *n* (%)				0.301
<260	127 (34)	89 (34)	38 (34)	
260–325	127 (34)	95 (36)	32 (29)	
>325	123 (33)	81 (31)	42 (38)	
Prothrombin time (s), *n* (%)				0.308
<11.1	143 (38)	94 (35)	49 (44)	
11.1–11.6	111 (29)	82 (31)	29 (26)	
>11.6	123 (33)	89 (34)	34 (30)	
Activated partial thromboplastin time (s), *n* (%)				0.273
<27.6	126 (33)	82 (31)	44 (39)	
27.6–31.9	128 (34)	92 (35)	36 (32)	
>31.9	123 (33)	91 (34)	32 (29)	
Thrombin time (s), *n* (%)				0.305
<14.5	131 (35)	87 (33)	44 (39)	
14.5–17.4	121 (32)	91 (34)	30 (27)	
>17.4	125 (33)	87 (33)	38 (34)	
Fibrinogen (g/L), *n* (%)				0.954
<2.68	130 (34)	91 (34)	39 (35)	
2.68–3.26	122 (32)	87 (33)	35 (31)	
>3.26	125 (33)	87 (33)	38 (34)	
Hemoglobin A1c (mmol/L), *n* (%)				0.423
<5.6	132 (35)	95 (36)	37 (33)	
5.6–6.2	133 (35)	88 (33)	45 (40)	
>6.2	112 (30)	82 (31)	30 (27)	
Total cholesterol (mmol/L), *n* (%)				0.874
<3.92	128 (34)	91 (34)	37 (33)	
3.92–4.97	124 (33)	85 (32)	39 (35)	
>4.97	125 (33)	89 (34)	36 (32)	
Triglycerides (mmol/L), n (%)				0.24
<1.00	128 (34)	88 (33)	40 (36)	
1.00–1.47	125 (33)	83 (31)	42 (38)	
>1.47	124 (33)	94 (35)	30 (27)	
High-density lipoprotein (mmol/L), n (%)				0.124
<1.02	129 (34)	96 (36)	33 (29)	
1.02–1.23	128 (34)	93 (35)	35 (31)	
>1.23	120 (32)	76 (29)	44 (39)	
Low-density lipoprotein (mmol/L), *n* (%)				0.707
<2.39	124 (33)	86 (32)	38 (34)	
2.39–3.23	127 (34)	87 (33)	40 (36)	
>3.23	126 (33)	92 (35)	34 (30)	
Very low-density lipoprotein (mmol/L), *n* (%)				0.983
<0.36	127 (34)	90 (34)	37 (33)	
0.36–0.54	126 (33)	88 (33)	38 (34)	
>0.54	124 (33)	87 (33)	37 (33)	
Apolipoprotein A1 (g/L), *n* (%)				0.083
<0 0.97	127 (34)	91 (34)	36 (32)	
0.97–1.12	128 (34)	97 (37)	31 (28)	
>1.12	122 (32)	77 (29)	45 (40)	
Apolipoprotein B100 (g/L), *n* (%)				0.982
<0.68	130 (34)	92 (35)	38 (34)	
0.68–0.88	125 (33)	88 (33)	37 (33)	
>0.88	122 (32)	85 (32)	37 (33)	
ApoA1 to ApoB100 ratio, *n* (%)				0.394
<1.19	126 (33)	94 (35)	32 (29)	
1.19–1.55	127 (34)	85 (32)	42 (38)	
>1.55	124 (33)	86 (32)	38 (34)	
Lipoprotein(a) (mg/L), *n* (%)				0.675
<121	126 (33)	89 (34)	37 (33)	
121–678	140 (37)	95 (36)	45 (40)	
>678	111 (29)	81 (31)	30 (27)	
Total protein (g/L), *n* (%)				0.361
<62	143 (38)	95 (36)	48 (43)	
62–67	125 (33)	93 (35)	32 (29)	
>67	109 (29)	77 (29)	32 (29)	
Albumin (g/L), *n* (%)				0.424
<35	159 (42)	110 (42)	49 (44)	
35–40	124 (33)	84 (32)	40 (36)	
>40	94 (25)	71 (27)	23 (21)	
Globulin (g/L), *n* (%)				0.272
<26	165 (44)	109 (41)	56 (50)	
26–28	106 (28)	77 (29)	29 (26)	
>28	106 (28)	79 (30)	27 (24)	
Albumin to globulin ratio, *n* (%)				0.653
<1.38	134 (36)	98 (37)	36 (32)	
1.38–1.56	119 (32)	81 (31)	38 (34)	
>1.56	124 (33)	86 (32)	38 (34)	
Homocysteine (μmol/L), *n* (%)				0.505
<15	135 (36)	94 (35)	41 (37)	
15–20	123 (33)	91 (34)	32 (29)	
>20	119 (32)	80 (30)	39 (35)	
Systemic immune-inflammatory index, *n* (%)				0.928
<515.27	126 (33)	89 (34)	37 (33)	
515.27–878.63	126 (33)	87 (33)	39 (35)	
>878.63	125 (33)	89 (34)	36 (32)	
Systemic inflammation response index, *n* (%)				0.761
<1.02	126 (33)	89 (34)	37 (33)	
1.02–1.75	126 (33)	91 (34)	35 (31)	
>1.75	125 (33)	85 (32)	40 (36)	
Neutrophil-to-lymphocyte ratio, *n* (%)				0.918
<2.36	126 (33)	87 (33)	39 (35)	
2.36–3.64	126 (33)	90 (34)	36 (32)	
>3.64	125 (33)	88 (33)	37 (33)	
Lymphocyte-to-monocyte ratio, *n* (%)				0.627
<2.86	126 (33)	92 (35)	34 (30)	
2.86–3.93	126 (33)	85 (32)	41 (37)	
>3.93	125 (33)	88 (33)	37 (33)	
Neutrophil-to-monocyte ratio, *n* (%)				0.942
<8.3	126 (33)	88 (33)	38 (34)	
8.3–10.0	125 (33)	87 (33)	38 (34)	
>10.0	126 (33)	90 (34)	36 (32)	
Neutrophil-to-HDL ratio, *n* (%)				0.411
<3.32	126 (33)	83 (31)	43 (38)	
3.32–4.90	126 (33)	91 (34)	35 (31)	
>4.90	125 (33)	91 (34)	34 (30)	
Lymphocyte-to-HDL ratio, *n* (%)				0.551
<1.10	126 (33)	91 (34)	35 (31)	
1.10–1.61	126 (33)	84 (32)	42 (38)	
>1.61	125 (33)	90 (34)	35 (31)	
Monocyte-to-HDL ratio, *n* (%)				0.551
<0.34	126 (33)	91 (34)	35 (31)	
0.34–0.51	126 (33)	84 (32)	42 (38)	
>0.51	125 (33)	90 (34)	35 (31)	
IL-6 (pg/mL), *n* (%)				0.533
<6.69	126 (33)	85 (32)	41 (37)	
6.69–12.99	126 (33)	93 (35)	33 (29)	
>12.99	125 (33)	87 (33)	38 (34)	
TNF-α (pg/mL), *n* (%)				0.294
<9.17	126 (33)	89 (34)	37 (33)	
9.17–16.90	126 (33)	94 (35)	32 (29)	
>16.90	125 (33)	82 (31)	43 (38)	
VCAM-1 (ng/mL), *n* (%)				0.302
<613.22	126 (33)	95 (36)	31 (28)	
613.22–873.64	126 (33)	86 (32)	40 (36)	
>873.64	125 (33)	84 (32)	41 (37)	
LP-PLA2 (ng/mL), *n* (%)				0.79
<203.60	126 (33)	90 (34)	36 (32)	
203.60–307.46	126 (33)	90 (34)	36 (32)	
>307.46	125 (33)	85 (32)	40 (36)	
CD40L (ng/mL), *n* (%)				0.536
<2.01	126 (33)	84 (32)	42 (38)	
2.01–4.54	126 (33)	90 (34)	36 (32)	
>4.54	125 (33)	91 (34)	34 (30)	
E-selectin (ng/mL), *n* (%)				0.409
<23.75	126 (33)	87 (33)	39 (35)	
23.75–48.32	126 (33)	94 (35)	32 (29)	
>48.32	125 (33)	84 (32)	41 (37)	
ADMA (μmol/L), *n* (%)				0.334
<0.52	126 (33)	83 (31)	43 (38)	
0.52–0.73	126 (33)	89 (34)	37 (33)	
>0.73	125 (33)	93 (35)	32 (29)	
vWF (%), *n* (%)				0.562
<97.47	126 (33)	85 (32)	41 (37)	
97.47–149.19	126 (33)	88 (33)	38 (34)	
>149.19	125 (33)	92 (35)	33 (29)	
ICAM-1 (ng/mL), *n* (%)				0.605
<182.04	130 (34)	88 (33)	42 (38)	
182.04–199.66	27 (7)	18 (7)	9 (8)	
>199.66	220 (58)	159 (60)	61 (54)	

This table presents a comprehensive comparison of potential predictive factors for cognitive impairment in a cohort of 377 patients with cerebral small vessel disease (CSVD). Divided into a training set comprising 265 patients for model development and a validation set consisting of 112 patients for efficacy assessment, the data encompass a range of clinical characteristics and inflammatory biomarkers. These include gender, smoking and drinking habits, hypertension, diabetes, hyperlipidemia, along with inflammatory markers like white blood cell count, platelet count, neutrophil count, and specific indicators closely related to CSVD such as IL-6, TNFα, and VCAM-1. The objective of this table is to demonstrate the comparability of the training and validation sets in these potential predictors, ensuring the generalizability and reliability of the predictive model’s outcomes. Statistical differences between the sets are indicated by *p*-values, with *p* < 0.05 denoting significant disparities.

### 3.2 Variable selection

**Figure 2:**
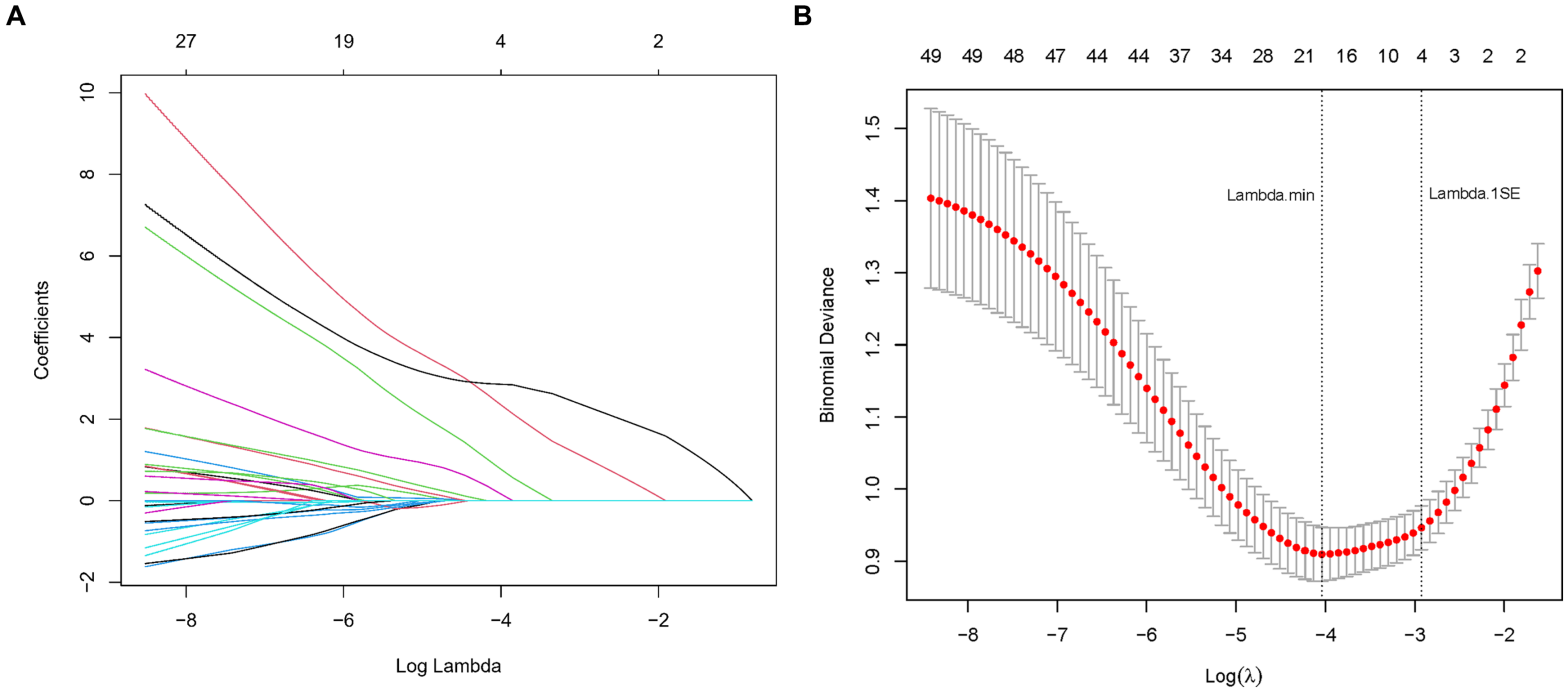
**(A)** LASSO coefficient profiles for CSVD cognitive impairment predictors. This figure shows the coefficient profiles of the predictors in the LASSO regression model. The *x*-axis represents the log of the regularization parameter (lambda), and the *y*-axis represents the coefficient values of the predictors. Each colored line represents a different predictor. As lambda increases (moving from left to right), the coefficients shrink towards zero, indicating the regularization effect of LASSO. The numbers at the top indicate the number of predictors included in the model for each lambda value. **(B)** Optimal lambda selection in LASSO model for CSVD cognitive impairment predictors. The graph demonstrates the selection of the optimal lambda value using cross-validation. The *x*-axis represents the log of lambda, and the *y*-axis represents the binomial deviance (a measure of model error). The red dots represent the mean binomial deviance for each lambda value, with error bars showing the standard error. The vertical dashed lines indicate the lambda values chosen by cross-validation: the left line represents the lambda that minimizes the binomial deviance, while the right line represents the largest lambda within one standard error of the minimum deviance, providing a more regularized and simpler model.

In our endeavor to elucidate the variables significantly associated with cognitive impairment in CSVD, we rigorously analyzed a dataset comprising 52 variables, which spanned demographic information, clinical history, and a wide array of laboratory measurements. Employing LASSO regression, a method recognized for its efficiency in variable selection and overfitting prevention, we utilized the glmnet package in R, integrating a 10-fold cross-validation technique to determine the optimal regularization parameter (*λ*). The selection of *λ* was guided by the one standard error rule from the minimum criterion in cross-validation error, ensuring the model’s parsimony without compromising its predictive accuracy (Figures 2A,B).

This meticulous process distilled the multitude of factors down to 4 pivotal indicators that bear a statistically significant relationship with cognitive impairment in CSVD patients. These indicators-Hypertension, CSVD Burden, ApoA1, and Age-demonstrated the strongest associations with cognitive impairment, underscoring their importance in the predictive model (Table 2). During the LASSO regression process, coefficients of less important variables shrink towards zero. We selected features with non-zero coefficients, which ensures that only the most relevant predictors are included in the final model. This approach balances model simplicity and predictive performance, enhancing both interpretability and robustness of the model. Although the coefficient for CSVD Burden is relatively small, it was retained due to its clinical significance in reflecting the overall severity of cerebral small vessel disease.

**Table 2:** Coefficients and lambda.1SE value of the LASSO regression.

Variable	Coefficients	Lambda.1SE
Hypertension	0.311	0.028
CSVD.Burden	0.0314	
ApoA1	−0.008	
Age	0.3131	

This table lists the coefficients and lambda.1SE values for key variables including hypertension, CSVD burden, ApoA1, and age in the LASSO regression model for cognitive impairment prediction in CSVD.

### 3.3 Multivariable analysis

Our multivariable logistic regression analysis, adjusting for potential confounders, identified significant associations between CSVD-related cognitive impairment and several key factors from the 12 variables initially pinpointed by LASSO regression. Notably, Hypertension (OR: 1.78, 95% CI: 1.32–3.84, *p* < 0.001) and Age (OR: 1.85, 95% CI: 1.55–3.55, *p* < 0.001) were potent predictors of impairment, indicating substantially elevated risks. A higher CSVD Burden also heightened the risk (OR: 1.83, 95% CI: 1.23–2.72, *p* = 0.003), whereas increased ApoA1 levels demonstrated a protective effect (OR: 0.64, 95% CI: 0.43–0.97, *p* = 0.034). These variables reflect critical demographic and biological aspects influencing CSVD prognosis, as detailed in Table 3.

**Table 3:** Binary logistic regression analysis.

	*B*	SE	OR	CI	*Z*	*p*-value
Hypertension	3.21	0.557	1.78	1.32–3.84	3.767	<0.001
CSVD.Burden	0.602	0.203	1.83	1.23–2.72	2.97	0.003
ApoA1	−0.443	0.209	0.64	0.43–0.97	−2.126	0.034
Age	3.758	0.656	1.85	1.55–3.55	3.732	<0.001

This table shows key predictors for cognitive impairment in CSVD, including regression coefficients, odds ratios, and their statistical significance.

### 3.4 Predictive model development

**Figure 3:**
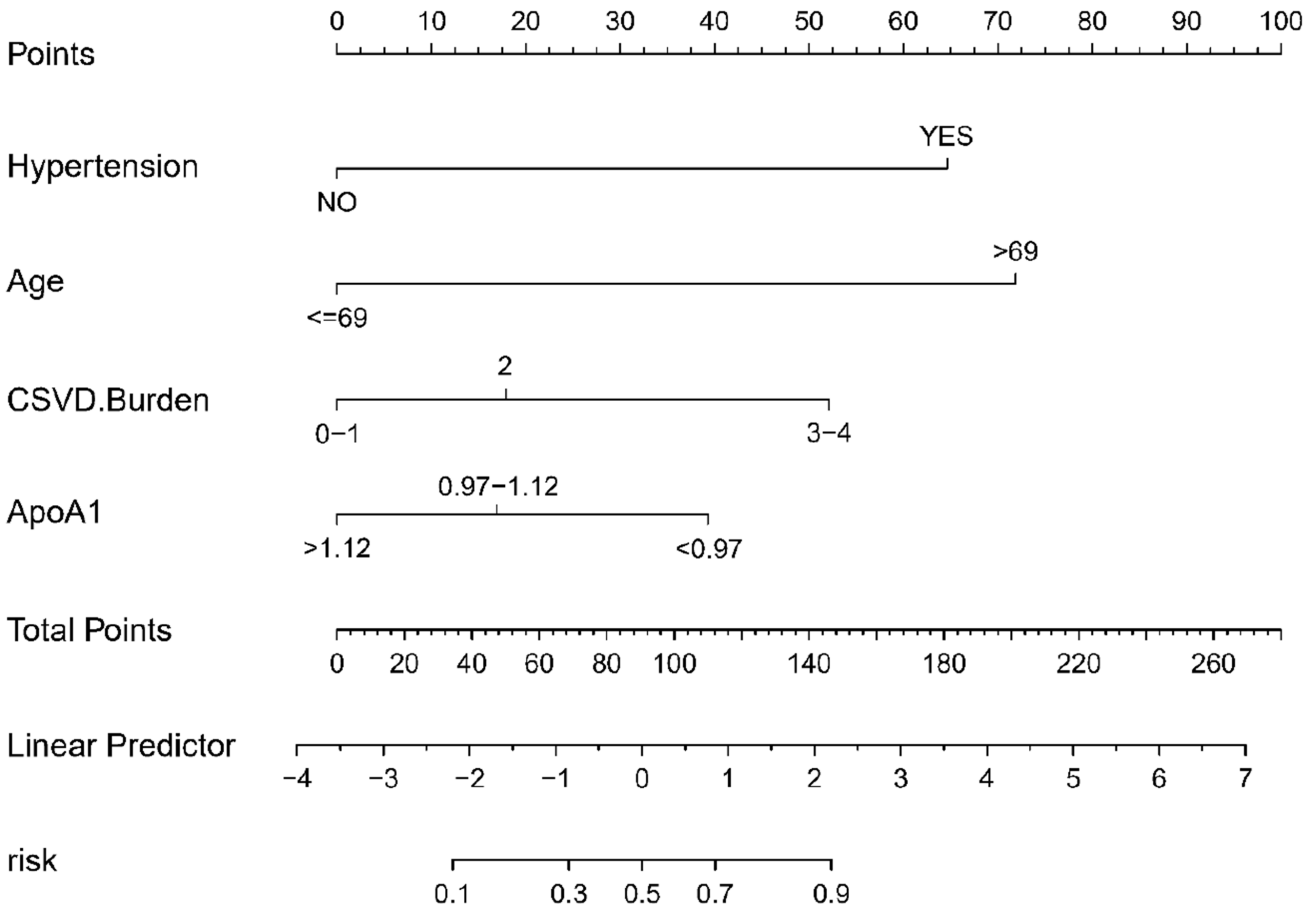
Nomogram for CSVD cognitive impairment risk prediction. This nomogram, based on logistic regression analysis, visually represents the contribution of each predictor to the overall risk of cognitive impairment in CSVD. Each predictor is assigned a score, and the total score corresponds to a probability of cognitive impairment. The predictors included are hypertension, CSVD burden, ApoA1 levels, and age.

In this study, a nomogram was developed to predict the probability of cognitive impairment in patients with CSVD, based on four identified predictive factors: Hypertension, CSVD Burden, ApoA1 levels, and Age (Figure 3). Each factor contributes to an individualized risk score, calculated using the nomogram, which correlates with the likelihood of cognitive decline. This tool provides clinicians with a concise and quantifiable method to assess risk and tailor patient management strategies effectively.

### 3.5 Evaluation of the predictive nomogram

**Figure 4:**
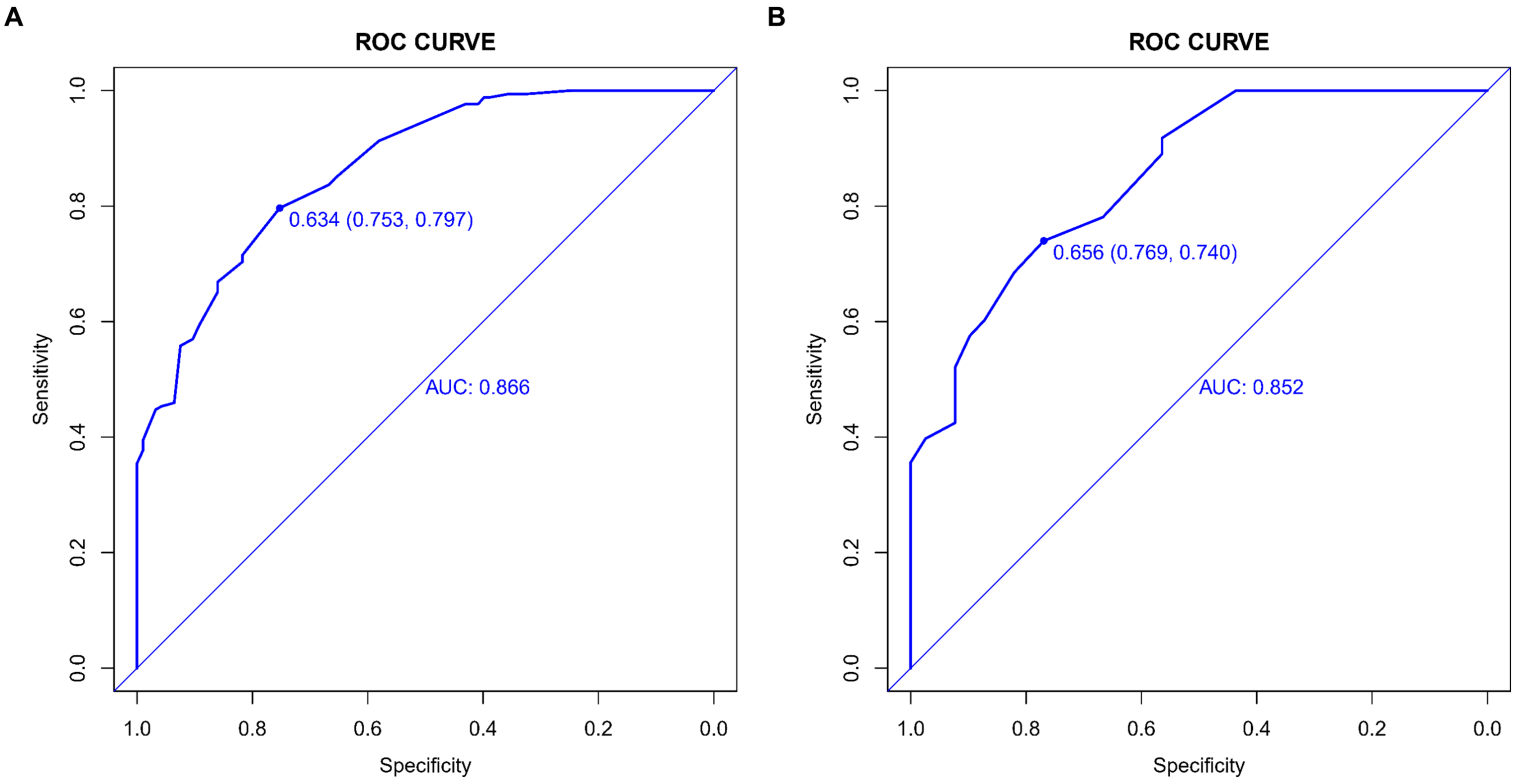
**(A,B)** ROC curves for CSVD cognitive impairment prediction model. These curves display the sensitivity and specificity of the model in both the training and validation sets, illustrating its diagnostic accuracy. The *x*-axis represents specificity, and the y-axis represents sensitivity. The area under the curve (AUC) values are shown for both sets, indicating the model’s performance. **(A)** ROC curve for the training set, showing the model’s performance with an AUC value of 0.866. **(B)** ROC curve for the validation set, showing the model’s performance with an AUC value of 0.852.

**Figure 5:**
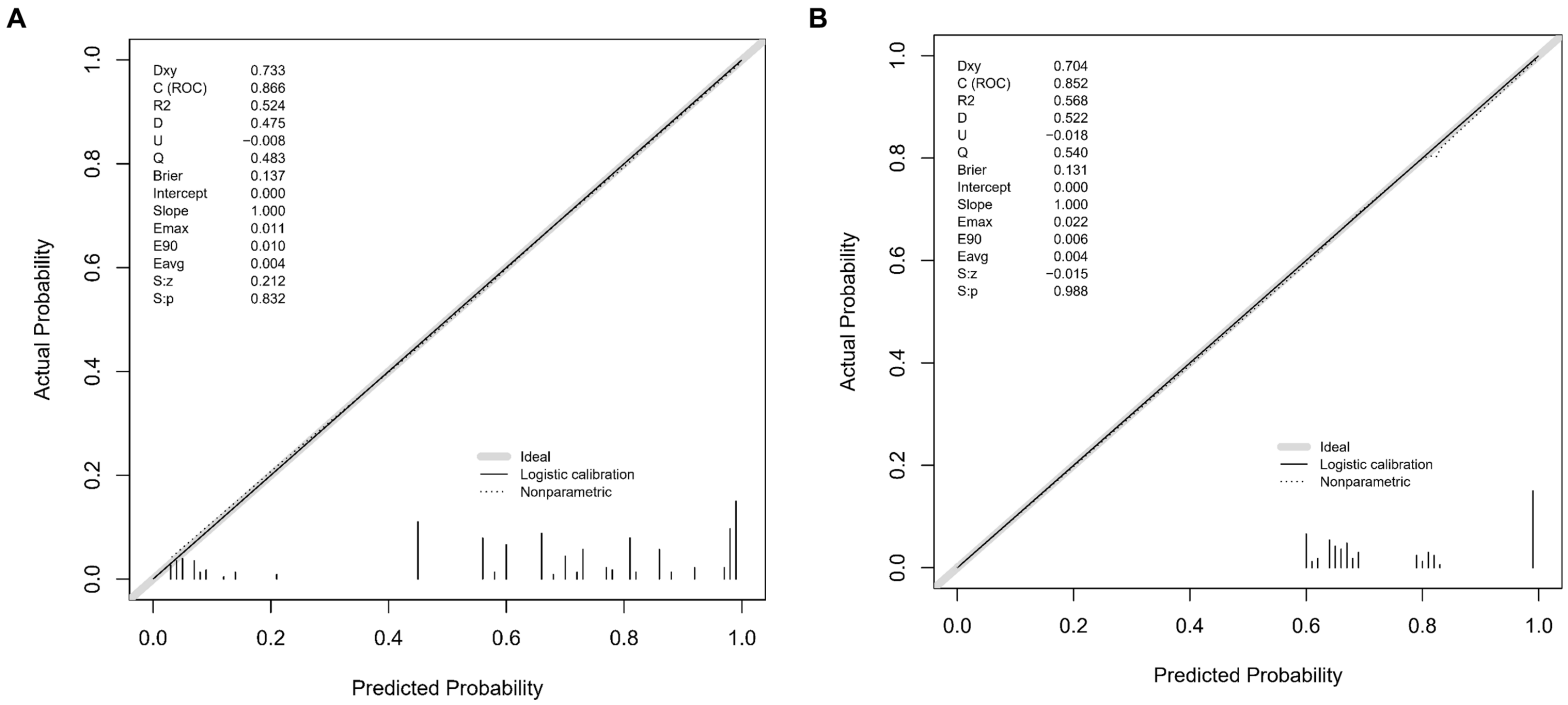
**(A,B)** Calibration plots for CSVD cognitive impairment prediction. These plots compare the predicted probabilities of cognitive impairment with the actual outcomes, demonstrating the model’s calibration accuracy in both the training and validation sets. The *x*-axis represents the predicted probability, and the *y*-axis represents the actual probability. The diagonal line represents perfect calibration, where predicted probabilities exactly match the actual outcomes. **(A)** Calibration plot for the training set. The plot shows how well the predicted probabilities agree with the actual probabilities in the training data. The grey line represents the ideal calibration, the solid black line shows the logistic calibration, and the dotted line represents the nonparametric calibration. **(B)** Calibration plot for the validation set. Similar to **A**, this plot shows the agreement between predicted and actual probabilities in the validation data. The grey line represents the ideal calibration, the solid black line shows the logistic calibration, and the dotted line represents the nonparametric calibration.

**Figure 6:**
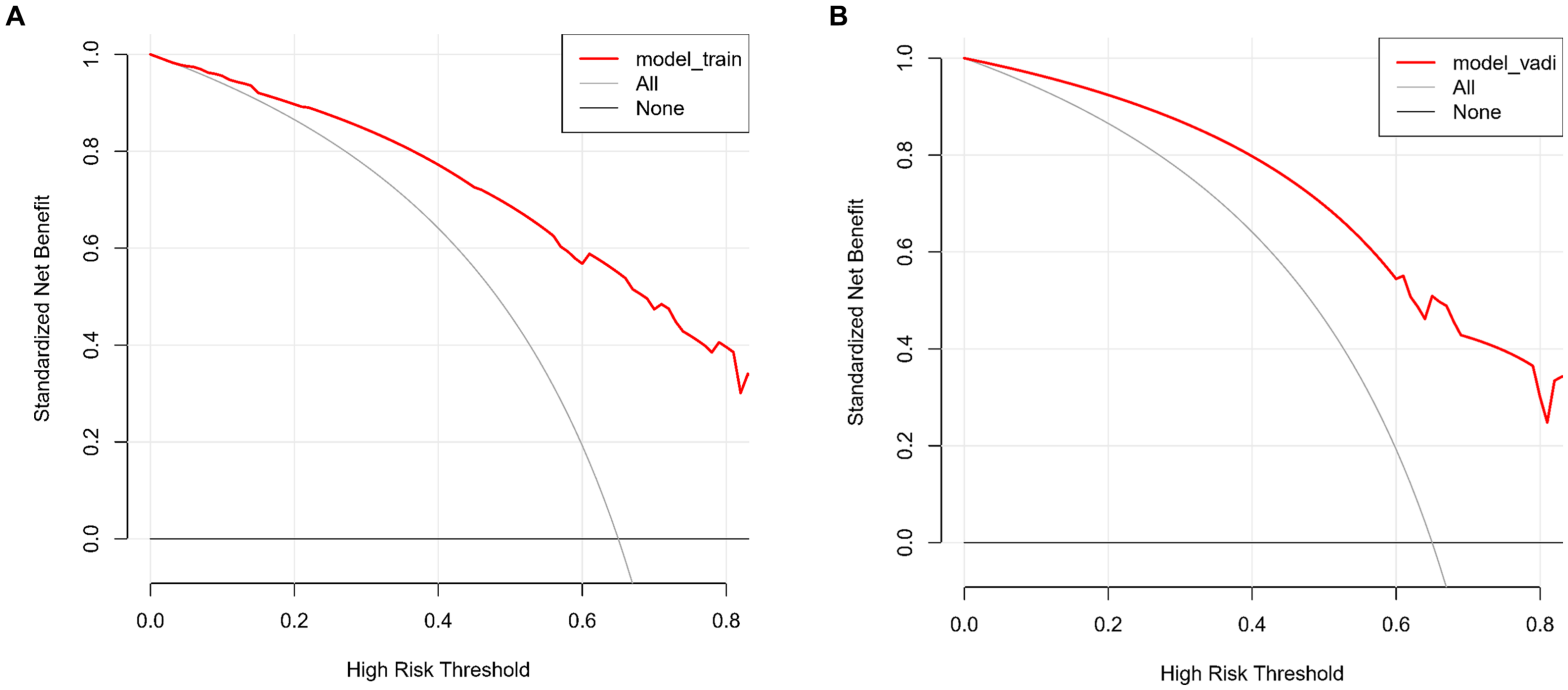
**(A,B)** Decision curve analysis for CSVD cognitive impairment model. These curves assess the clinical usefulness of the model by comparing the net benefits of different treatment strategies in both the training and validation sets. The *x*-axis represents the high-risk threshold, and the *y*-axis represents the standardized net benefit. **(A)** Decision curve for the training set. The red line represents the net benefit of the predictive model for the training data. The grey line, which slopes downward, represents the net benefit assuming all patients are treated. The black line, which is horizontal, represents the net benefit assuming no patients are treated. **(B)** Decision curve for the validation set. Similar to **A**, the red line represents the net benefit of the predictive model for the validation data. The grey line, which slopes downward, represents the net benefit assuming all patients are treated. The black line, which is horizontal, represents the net benefit assuming no patients are treated.

The predictive accuracy of our CSVD cognitive impairment nomogram was assessed using the area under the receiver operating characteristic (AUC-ROC) curve, with the training set demonstrating an AUC of 0.866 (95% CI: 0.823–0.909), and the validation set showing an AUC of 0.852 (95% CI: 0.781–0.923). These results indicate excellent discriminative ability across both datasets (Figures 4A,B). Calibration curves closely align with the ideal line in both training (Figure 5A) and validation (Figure 5B) sets, suggesting that the nomogram’s predicted probabilities of cognitive impairment are accurate. Decision curve analysis (DCA) for both datasets confirms the model’s clinical usefulness, with the net benefit substantially outweighing the treat-all or treat-none strategies (Figures 6A,B). In application, the nomogram showed high sensitivity and specificity, further evidencing its robustness. In the training set, the model exhibited a sensitivity of 75.3% and a specificity of 79.7% (Figure 4A). In contrast, in the validation set, the model showed a sensitivity of 76.9% and a specificity of 74.0% (Figure 4B). For the training set, the nomogram achieved a PPV of 87.32% and an NPV of 63.47%, and for the validation set, the PPV was 84.60% and NPV was 63.30%. These additional metrics further reinforce the nomogram’s strong and consistent performance, advocating its potential utility in clinical practice for risk stratification and management of CSVD-associated cognitive impairment.

## 4 Discussion

The development of our predictive model for cognitive impairment in CSVD is a significant stride in neurovascular research, bridging a gap in early diagnostic methodologies. By integrating a diverse range of variables-demographic, clinical, neuroimaging, and biomarkers-our study sheds new light on the intricate relationship between CSVD and cognitive decline. This approach not only enhances our understanding of CSVD’s impact on cognitive functions but also introduces a valuable tool for early detection and intervention, marking a crucial step forward in addressing this complex condition.

Our study contributes to the evolving landscape of CSVD research by presenting a comprehensive predictive model for cognitive impairment. This model integrates a wide array of variables, akin to the multifaceted approaches seen in recent literature. The deep learning approach of Duan et al. (14) in segmenting CSVD features on imaging contrasts with our model that combines clinical, biochemical, and imaging data for a more rounded prediction. Egle et al. (15) emphasized diffusion tensor imaging’s role in predicting dementia, a perspective that complements our model’s inclusion of imaging alongside other clinical variables. Our work resonates with the findings of Jiménez-Balado et al. (16) regarding the predictive value of blood pressure monitoring in CSVD. However, our approach is more comprehensive, encompassing a broader range of indicators. Similarly, while Li et al. (17) explored machine learning models for dementia prediction in CSVD, our study adds to this by leveraging both advanced statistical and machine learning techniques, emphasizing the integration of diverse data types. Furthermore, Liu et al. (9) highlighted the combination of Aβ42 levels and total CSVD scores in predicting cognitive impairment. Our model expands upon this by integrating these biomarkers into a broader predictive framework. Lastly, van Uden et al. (18) and Zhang et al. (10) focused on specific imaging and clinical parameters. Our research builds upon these studies by offering a more comprehensive model that incorporates their insights into a broader predictive framework. In summary, our study not only aligns with current research trends in CSVD but also extends them by providing a more holistic approach to the prediction of cognitive impairment. This comprehensive model could significantly enhance clinical decision-making and patient management in CSVD.

Age is acknowledged as a critical predictor for the progression of CSVD and associated cognitive impairments. The study by Hamilton et al. (19) corroborates this, demonstrating a significant correlation between the total burden of CSVD and a decline in cognitive abilities in the elderly. This finding not only validates the importance of age as a predictive factor but also underscores the heightened risk for cognitive health deterioration with advancing age. Hypertension is a primary contributor to the development of CSVD and subsequent cognitive decline. The research conducted by Amier et al. (20) revealed a substantial association between markers of hypertensive exposure, as evident in cardiovascular MRI, and both CSVD and cognitive impairments. Additionally, the study by Hainsworth et al. (21) accentuates the close link between hypertension and small vessel disease, aligning well with our findings and underscoring the necessity to consider hypertension’s role in clinical predictive models. The imaging burden of CSVD is significantly associated with declines in cognitive function. Hosoya et al. (22) observed an independent association between imaging markers of CSVD and reductions in global cognitive function and attention. Our research echoes this observation, highlighting the crucial role of CSVD imaging burden in predicting cognitive impairments. Our study further identifies ApoA1 as a novel negative predictor for cognitive impairment in CSVD, aligning with emerging research in neurodegenerative conditions. Studies by Choi et al. (23) and Slot et al. (24) reported similar associations between ApoA1 levels and cognitive decline, suggesting ApoA1’s potential role in amyloid-independent neurodegeneration. Moreover, research by Das et al. (25) and Deng et al. (26) underscore ApoA1’s neuroprotective effects in Parkinson’s disease, while Rao et al. (27) highlight the complex interaction between ApoA1 levels and genetic factors in cognitive impairment. These findings collectively corroborate our results, emphasizing ApoA1’s significance in diagnosing and treating CSVD-related cognitive impairment.

In our study, we have elucidated several pivotal findings that hold significant implications for understanding the development of cognitive impairment in the context of CSVD. Firstly, we have reaffirmed hypertension and advanced age as the principal risk factors for cognitive impairment in CSVD. Both factors exhibit a substantial association with an increased risk of cognitive impairment, underscoring the imperative need for clinicians to closely monitor cognitive functions in patients exhibiting these characteristics. Additionally, our model underscores the critical importance of the cumulative burden of CSVD-related lesions (CSVD burden) and serum levels of apolipoprotein A1 (ApoA1) in predicting cognitive function. These findings provide valuable clinical cues to assist physicians in identifying high-risk individuals and devising tailored management strategies.

The results of our investigation hold profound clinical utility. The predictive model we have developed, based on CSVD risk factors, serves as a valuable tool for the early identification of individuals at risk of developing cognitive impairment. This not only facilitates timely intervention and treatment but also has the potential to enhance the quality of life for affected individuals. Our model offers a personalized risk assessment tool that guides clinicians in formulating precise treatment plans, while also empowering patients with information about their individual risks. Despite the significant achievements of our study, several avenues for future research merit exploration. Firstly, we advocate for further research into the biomarkers of CSVD-related cognitive impairment to refine risk prediction. Furthermore, as neuroimaging and molecular biology techniques continue to advance, we encourage the integration of these advanced technologies into a more comprehensive predictive model for CSVD. Additionally, longitudinal studies with extended follow-up periods are warranted to gain deeper insights into the progression and trajectory of cognitive impairment in CSVD.

Our study is not without limitations. Firstly, its cross-sectional design precludes the establishment of causal relationships. Although our model demonstrates excellent predictive performance for CSVD-related cognitive impairment, further validation in larger multicenter cohorts is warranted. Additionally, the potential for selection bias may exist, as all participants were sourced from a single hospital. Despite the inclusion of a comprehensive array of clinical, biochemical, and imaging data, there may be other unexplored factors that influence the risk of cognitive impairment in CSVD. Moreover, the sample size of this study is relatively small. Future research should include a larger sample size to enhance the reliability and generalizability of the findings.

In summary, our study presents a comprehensive predictive model that holds significant promise in forecasting the risk of cognitive impairment in the context of CSVD. This model integrates clinical, biochemical, and imaging data, furnishing clinicians with a powerful tool to identify high-risk individuals and devise personalized management strategies. While limitations and avenues for future research exist, we believe that this study marks a pivotal advancement in the early diagnosis and intervention of CSVD-related cognitive impairment.

## Data availability statement

The original contributions presented in the study are included in the article/supplementary material, further inquiries can be directed to the corresponding author.

## Ethics statement

The studies involving humans were approved by the Ethics Committee of the Affiliated Hospital of Hebei University. The committee is affiliated with Hebei University, located in Baoding, China. The studies were conducted in accordance with the local legislation and institutional requirements. The participants provided their written informed consent to participate in this study.

## Author contributions

NL: Conceptualization, Data curation, Formal analysis, Funding acquisition, Investigation, Methodology, Resources, Software, Validation, Visualization, Writing – original draft, Writing – review & editing. YG: Conceptualization, Data curation, Formal analysis, Investigation, Methodology, Resources, Validation, Visualization, Writing – original draft, Writing – review & editing. L-tL: Conceptualization, Formal analysis, Methodology, Software, Supervision, Writing – review & editing. Y-dH: Conceptualization, Data curation, Formal analysis, Investigation, Methodology, Validation, Visualization, Writing – review & editing. LL: Investigation, Methodology, Validation, Writing – review & editing. NJ: Data curation, Software, Writing – review & editing. Y-jC: Formal analysis, Software, Writing – review & editing. Y-nM: Investigation, Methodology, Writing – review & editing. YJ: Conceptualization, Formal analysis, Investigation, Project administration, Resources, Software, Supervision, Validation, Visualization, Writing – original draft, Writing – review & editing.

## Footnotes

^1^ http://www.r-project.org/
